# A sexually dimorphic signature of activity-dependent BDNF signaling on the intrinsic excitability of pyramidal neurons in the prefrontal cortex

**DOI:** 10.3389/fncel.2024.1496930

**Published:** 2024-11-06

**Authors:** Kaijie Ma, Daoqi Zhang, Kylee McDaniel, Maria Webb, Samuel S. Newton, Francis S. Lee, Luye Qin

**Affiliations:** ^1^Division of Basic Biomedical Sciences, Sanford School of Medicine, University of South Dakota, Vermillion, SD, United States; ^2^Department of Biotechnology, Mount Marty University, Yankton, SD, United States; ^3^School of Health Sciences, University of South Dakota, Vermillion, SD, United States; ^4^Department of Psychiatry, Department of Pharmacology, Sackler Institute for Developmental Psychobiology, Weill Cornell Medicine, New York, NY, United States

**Keywords:** activity-dependent neural signaling, BDNF, Val66Met polymorphism, intrinsic neuronal excitability, sex

## Abstract

Autism spectrum disorder (ASD) is a group of neurodevelopmental disorders with strong genetic heterogeneity and more prevalent in males than females. We and others hypothesize that diminished activity-dependent neural signaling is a common molecular pathway dysregulated in ASD caused by diverse genetic mutations. Brain-derived neurotrophic factor (BDNF) is a key growth factor mediating activity-dependent neural signaling in the brain. A common single nucleotide polymorphism (SNP) in the pro-domain of the human *BDNF* gene that leads to a methionine (Met) substitution for valine (Val) at codon 66 (Val66Met) significantly decreases activity-dependent BDNF release without affecting basal BDNF secretion. By using mice with genetic knock-in of this human BDNF methionine (Met) allele, our previous studies have shown differential severity of autism-like social deficits in male and female BDNF^+/Met^ mice. Pyramidal neurons are the principal neurons in the prefrontal cortex (PFC), a key brain region for social behaviors. Here, we investigated the impact of diminished activity-dependent BDNF signaling on the intrinsic excitability of pyramidal neurons in the PFC. Surprisingly, diminished activity-dependent BDNF signaling significantly increased the intrinsic excitability of pyramidal neurons in male mice, but not in female mice. Notably, significantly decreased thresholds of action potentials were observed in male BDNF^+/Met^ mice, but not in female BDNF^+/Met^ mice. Voltage-clamp recordings revealed that the sodium current densities were significantly increased in the pyramidal neurons of male BDNF^+/Met^ mice, which were mediated by increased transcriptional level of *Scn2a* encoding sodium channel Na_V_ 1.2. Medium after hyperpolarization (mAHP), another important parameter to determine intrinsic neuronal excitability, is strongly associated with neuronal firing frequency. Further, the amplitudes of mAHP were significantly decreased in male BDNF^+/Met^ mice only, which were mediated by the downregulation of *Kcnn2* encoding small conductance calcium-activated potassium channel 2 (SK2). This study reveals a sexually dimorphic signature of diminished activity-dependent BDNF signaling on the intrinsic neuronal excitability of pyramidal neurons in the PFC, which provides possible cellular and molecular mechanisms underpinning the sex differences in idiopathic ASD patients and human autism victims who carry BDNF Val66Met SNP.

## 1 Introduction

ASD is highly genetically heterogeneous and caused by both inherited and *de novo* gene mutations (Simons Foundation Autism Research Initiative, SFARI) (Satterstrom et al., 2020; Homozygosity Mapping Collaborative for Autism et al., 2014). We and others hypothesize that diminished activity-dependent neural signaling is a common molecular pathway dysregulated in ASD caused by diverse genetic mutations (Ebert and Greenberg, 2013; Yap and Greenberg, 2018). Brain-derived neurotrophic factor (BDNF) is synthesized and secreted in response to neuronal activity, which is a key molecule mediating activity-dependent neural signaling and has particular roles in synaptic transmission, synaptic connections, neurotransmitter release, and synaptic plasticity during postnatal neuronal development (Song et al., 2017; Greenberg et al., 2009; Chao, 2003). A common single nucleotide polymorphism (SNP) in the pro-domain of the human *BDNF* gene that leads to a methionine (Met) substitution for valine (Val) at amino acid 66 (Val66Met) significantly reduces dendritic trafficking, synaptic localization of the protein, and decreases up to 30% of activity-dependent BDNF release without affecting basal BDNF secretion (Chen et al., 2006; Egan et al., 2003). This human BDNF Val66Met SNP has been linked to psychiatric diseases, including anxiety (Chen et al., 2006; Egan et al., 2003; Notaras et al., 2015). Human studies showed that the BDNF Val66Met SNP was significantly associated with children with ASD in the Korean population (Yoo et al., 2014). Abnormal cortical developmental was observed in autism children harboring BDNF Val66Met SNP (Raznahan et al., 2009). By using mice with genetic knock-in of this human BDNF methionine (Met) allele, our recent studies have shown that diminished activity-dependent BDNF signaling differentially induces autism-like social deficits in males and females, and that males appear to be more severe than females (Ma et al., 2023).

In humans and rodents, the prefrontal cortex (PFC) is a hub brain region critical for “high-level” executive functions, including social behavior and cognition (Arnsten and Rubia, 2012; Davidson, 2002; Qin et al., 2019; Qin et al., 2018). Ours and others’ studies have demonstrated that *Shank3*-deficiency significantly diminished NMDA receptors- and AMPA (*α*-amino-3-hydroxy-5-methyl-4-isoxazolepropionic acid) receptors-mediated glutamatergic synaptic transmission in the PFC, which caused social deficits in mouse models (Duffney et al., 2015; Wang et al., 2016; Qin et al., 2018). Increased neural activity in the PFC is a key pathogenesis of social deficits in ASD (Lee et al., 2017; Gao and Penzes, 2015; Yizhar et al., 2011; Spratt et al., 2019). Pyramidal neurons are the principal neurons in the PFC, which send out long-distance glutamatergic excitation to the subcortical regions (Murugan et al., 2017; Zhong et al., 2022; Zhong et al., 2020). Intrinsic neuronal excitability is the capability of a neuron to generate action potentials in response to integrative synaptic inputs such as somatic current injections, which is determined primarily by the densities and functions of voltage-gated ion channels (Zhang and Linden, 2003). The changes in intrinsic neuronal excitability are thought to play an important role in learning and memory (Chen et al., 2020), and social deficits in autism mouse models (Spratt et al., 2019; Zhang et al., 2021; Tatsukawa et al., 2019; Eaton et al., 2021; Spratt et al., 2021; Khandelwal et al., 2021). However, little is known about the impact of activity-dependent BDNF signaling on the intrinsic neuronal excitability of pyramidal neurons in the PFC.

According to the latest data from the Autism and Developmental Disabilities Monitoring (ADDM) network (CDC: Centers for Diseases Control and Prevention), one in 36 8-year-old children have been identified with ASD in 2020. Males are four times more likely to be diagnosed with ASD than females. To highlight the need to investigate the neural mechanisms of ASD in both sexes (Shansky and Woolley, 2016), in this study, we characterized the impact of diminished activity-dependent BDNF signaling on the intrinsic neuronal excitability of pyramidal neurons in the PFC by using male and female BDNF^+/+^ and BDNF^+/Met^ mice.

## 2 Materials and methods

### 2.1 Animal care and husbandry

The use of animals and procedures performed were approved by the Institutional Animal Care and Use Committee of Sanford School of Medicine, University of South Dakota. A mouse model with genetic knock-in of a human BDNF Met variant was created, and the procedures for heterozygote breeding and genotyping were described previously (Chen et al., 2006). These mice were backcrossed more than 12 generations into the C57BL/6 strain. Animals were group-housed (*n* = 4–5) in standard cages and were kept on a 12-h light–dark cycle in a temperature-controlled room. Food and water were available *ad libitum*. Experiments were performed in male and female BDNF^+/Met^ mice and sex- and age- matched WT littermates BDNF*^+/+^*, which were derived from heterozygous BDNF^+/Met^ breeding pairs.

### 2.2 Brain slice preparation

Coronal brain slices containing PFC were prepared from 2 months old male and female BDNF*^+/+^* and BDNF^+/Met^ mice as described previously (Qin et al., 2019; Qin et al., 2018; Qin et al., 2021). In brief, mice were anesthetized with isoflurane and rapidly decapitated. Brains were immediately removed, iced, and cut into 300 μm slices by a Vibratome (Leica VP1000S, Leica Microsystems Inc.). Brain slices were then incubated at 33°C in artificial cerebrospinal fluid (ACSF) (in mM: 130 NaCl, 26 NaHCO3, 3 KCl, 5 MgCl2, 1.25 NaH2PO4, 1 CaCl2, 10 glucose, pH 7.4, 300 mOsm) for 1 h and then kept for 1–4 h at room temperature (20–21°C) bubbling with 95% O2, 5% CO2.

### 2.3 Whole cell patch-clamp recordings

For recordings, the brain slice was positioned in a perfusion chamber attached to the fixed stage of an upright microscope (Olympus) and submerged in continuously flowing oxygenated ACSF (in mM: 130 NaCl, 26 NaHCO3, 1 CaCl2, 5 MgCl2, 3 KCl, 1.25 NaH2PO4, 10 glucose, pH 7.4, 300 mOsm). Layer V pyramidal neurons in the PFC were visualized with infrared differential interference contrast video microscopy. Recordings were performed with a multi-Clamp 700B amplifier (Molecular Devices), and data were acquired using pClamp 11.2 software, filtered at 1 kHz and sampling rate at 10 kHz with an Axon Digidata 1550B plus HumSilencer digitizer (Molecular Devices). Recording electrodes were pulled from borosilicate glass capillaries (1.5/0.86 mm OD/ID) with a micropipette puller (Sutter Instrument, model P-97, Novato, CA). The resistances of patch electrodes were 4–6 MΩ when filled with internal solution.

Whole-cell current-clamp recordings were used to measure action potentials. The brain slice was bathed in a modified ACSF with low (0.5 mM) MgCl2 to elevate neuronal activity, which more closely mimics the ionic composition of the brain interstitial fluid *in situ*. AMPA (*α*-amino-3-hydroxy-5-methyl-4-isoxazolepropionic acid) (20 mM CNQX) and GABA_A_ (g-aminobutyric acid) receptors blockers (20 mM bicuculline) were added in action potentials recordings as in our previous studies (Qin et al., 2021). The pipettes were filled with an intracellular solution (in mM): 124 K-gluconate, 1 MgCl2, 6 KCl, 5 EGTA, 10 HEPES, 0.5 CaCl2, and 12 phosphocreatine, 5 MgATP, 0.5 Na2GTP, 0.2 Leupeptin, pH 7.2–7.3, 265–270 mOsm. To label the neurons under recording, the internal solution was supplemented with 0.05% sulforhodamine B. Slices were then fixed with 4% paraformaldehyde for 30 min, washed 3 times in PBS (pH 7.4). The neuronal images were acquired with a Leica TCS SP8 confocal microscope.

Seal formation and membrane rupture were done in a voltage-clamp mode at holding potential of −70 mV. Resting membrane potentials were measured immediately on break-in. A series of 250 ms current pulses (from −20 to 130 pA, 10 pA increments) were elicited to obtain action potential firing trains while the neurons were held at a fixed potential of −70 mV.

Rheobase was the minimal electric current required to elicit an action potential when current was injected into a neuron holding at −70 mV. The first spike latency was the time interval between the beginning of the current step and the occurrence of the first action potential elicited by minimal electric current. The voltage threshold of action potential was defined as the voltage where the value of dV/dt first exceeded 10 mV/ms at the first spike elicited by minimal electric current. The amplitude of medial after hyperpolarization (mAHP) was measured as the difference between the threshold and the peak of the most negative followed the action potentials after the first action potential (regular spiking neurons) or after short-bursts (intrinsic bursting neurons) elicited by 100 pA current injection (Wu et al., 2016).

The input resistance (r) was determined by injecting a − 100 pA, 250 ms hyperpolarizing current into the neuron holding at −70 mV. The membrane time constant (τ) was calculated using a single exponential fit of the voltage change in response to −100 pA hyperpolarizing current injection with 250 ms duration. The cell capacitance (c) was calculated under a current-clamp mode using the formula c = r/τ, where c was membrane capacitance, r was cell membrane resistance, and τ was membrane time constant (Sun et al., 2020).

Whole-cell voltage-clamp recordings were used to measure sodium currents. The brain slice was bathed in ACSF. Recording pipette contained the following internal solution (in mM: 100 CsCl, 10 tetraethylammonium chloride (TEA-Cl), 5 4-aminopyridine (4-AP), 10 HEPES, 4 NaCl, 1 MgCl2, 5 EGTA, 12 phosphocreatine, 5 MgATP, 0.5 Na2GTP, 0.2 Leupeptin, pH 7.2–7.3, 265–270 mOsm) (Milescu et al., 2010). Neurons were held at −70 mV and stepped to a range of potentials (−70 to +50 mV, 10 mV increments) for 100 ms each. Current densities (current/capacitance) were plotted as a function of depolarizing potential to generate current densities-voltage curves. All electrophysiological recordings were performed at room temperature (21–22°C). During recordings, neurons with leak currents >200 pA were discarded. Series resistance (Rs) was compensated to 80–90%. Cells having series resistance >10 MΩ or change above 20% throughout the experiments were excluded from analysis (Sontheimer and Ransom, 2002; Manz et al., 2021). The leakage current amplitudes were subtracted offline from the current peaks, and the capacitive transients were not cancelled (Surges et al., 2006). The amplitude of sodium current was measured as the difference between the onset of depolarization (after capacitive transient) and the peak of inward current.

### 2.4 Quantitative real-time RT-PCR

Total RNA was isolated from mouse PFC punches using Trizol reagent (Invitrogen) and treated with DNase I (Invitrogen) to remove genomic DNA. Then the iScriptTM cDNA synthesis Kit (Bio-Rad) was used to obtain cDNA from the tissue mRNA. Quantitative real time PCR was carried out using the iCycler iQ™ RealTime PCR Detection System and iQ™ Supermix (Bio-Rad) according to the manufacturer’s instructions. In brief, GAPDH was used as the housekeeping gene for quantitation of the expression of target genes in samples from male and female BDNF^+/+^ and BDNF^+/Met^ mice. Fold changes in the target genes were determined by: Fold change = 2^-∆(∆CT)^, where ∆CT = C_T_ (target) - C_T_(GAPDH), and ∆(∆C_T_) = ∆C_T_ (another group) - ∆C_T_ (male BDNF^+/+^). C_T_ (threshold cycle) is defined as the fractional cycle number at which the fluorescence reaches 10X of the standard deviation of the baseline. A total reaction mixture of 20 mL was amplified in a 96-well thin-wall PCR plate (Bio-Rad) using the following PCR cycling parameters: 95°C for 5 min followed by 40 cycles of 95°C for 45 s, 55°C for 45 s, and 72°C for 45 s. Primers for all target genes are listed in Table 1.

**Table 1:** List of primers used in qPCR experiments.

Target Gene	Forward	Reverse	Gene reference
*Gapdh*	gacaactcactcaagattgtcag	atggcatggactgtggtcatgag	NM_001289726.1
*Scn1a*	agcttcaacttcttcaccag	tgggccattttcatcatcat	NM_001313997.1
*Scn2a*	ccttgctgctattgaacaac	cctgcttccaagtcactatt	NM_001099298.3
*Scn3a*	agaatctcttgctgctatcg	agcttccaagtcactgtttg	NM_001355166.1
*Scn8a*	ctttcatctacggggacatc	gcgctaaatctgaagagagt	NM_001077499.2
*Hcn1*	ggtcaacaaattctccctcc	agtcactgtacggatggata	NM_010408.3
*Hcn2*	cccaaggtttcgttctcat	aattggcgctgcaggaag	NM_008226.2
*Hcn3*	tactgggatctcatcatgct	cagagaggacattgaagacg	NM_008227.2
*Hcn4*	gtcagcagggttttggatta	cacgggtatgatgatcagatt	NM_001081192.3
*Kcnn1*	atacaccaaggagtcactct	caagaacagctggatctctc	NM_001363407.2
*Kcnn2*	aacagctctgacatggaaac	cttgtcctggctctgttg	NM_001312905.2
*Kcnn3*	ctggtctgttgcactcttc	tggtcattgagatttagctgg	NM_080466.2
*Kcnn4*	ctgagatgttgtggttcctg	ccacaataagacaaaggagga	NM_008433.5
*Kcnq2*	ttagtcttctcctgccttgt	cacaaagtactcaacaccga	NM_010611.3
*Kcnq3*	aagtcaccttggcgctag	gttgttcctcttgactggg	NM_152923.3
*Kcnq4*	tgtctgtactgtccaccat	atatactccaagccaaagacc	NM_001081142.3
*Kcnq5*	ctgtacaacgtgctggag	ttttgtatgctcagggatgg	NM_001160139.1

### 2.5 Statistical analysis

Data were analyzed with GraphPad Prism 10 (GraphPad) and Clampfit 11.2 (Molecular Devices, Sunnyvale, CA). For statistical significance, experiments with more than two groups were assessed with two-way or three-way ANOVA, followed by *post hoc* Bonferroni tests for multiple comparisons. All values were presented as mean ± SEM. *p* < 0.05 was considered statistically different.

## 3 Results

### 3.1 Diminished activity-dependent BDNF signaling significantly increases the intrinsic excitability of pyramidal neurons in the PFC of male mice, but not in female mice

**Figure 1:**
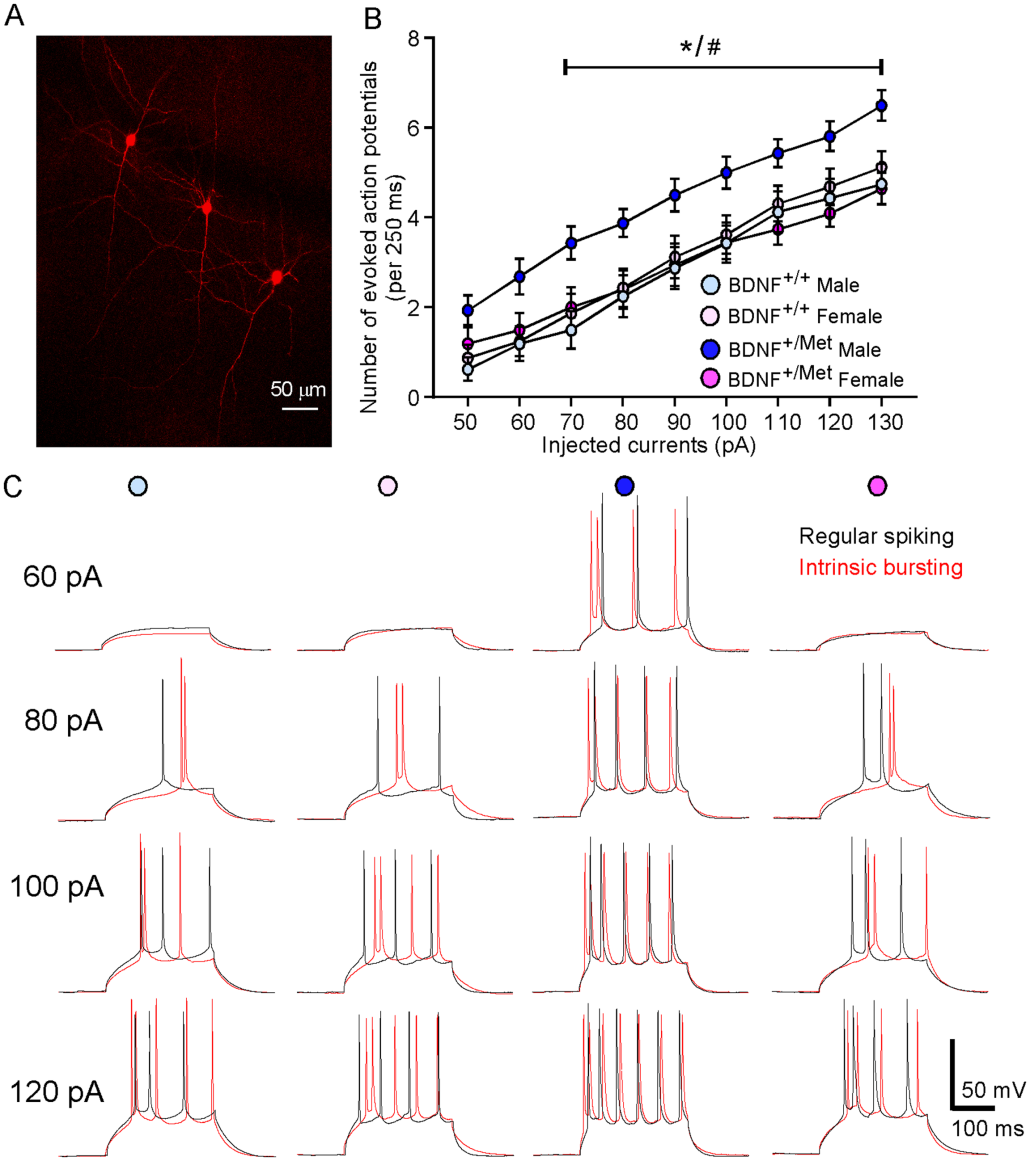
Diminished activity-dependent BDNF signaling significantly increases intrinsic neuronal excitability of pyramidal neurons in the PFC of male mice, but not in female mice. **(A)** A representative confocal image showing layer V pyramidal neurons used for recording. **(B)** Quantification of the number of evoked action potentials in response to injected currents in the pyramidal neurons of PFC from BDNF^+/+^ and BDNF^+/Met^ mice. *n* = 16–20 neurons/4–5 mice/group. **(C)** Representative action potential traces from regular spiking (black) and intrinsic bursting (red) pyramidal neurons. *p < 0.05, BDNF^+/Met^ versus BDNF^+/+^; #*p* < 0.05, male versus female, three-way ANOVA.

To examine the impact of diminished activity-dependent BDNF signaling on the intrinsic excitability of pyramidal neurons in the PFC, we performed a whole-cell patch clamp to evoke action potentials by injecting a series of constant currents while holding the membrane potential at -70 mV. Given that deep layer glutamatergic pyramidal neurons in PFC showed the clearest deficits in autistic children (Stoner et al., 2014), pyramidal neurons in layer V were selected for electrophysiological measurements. As shown in Figure 1A, pyramidal neurons can be reliably identified by electrophysiological recordings according to our previous studies (Qin et al., 2018; Qin et al., 2021). Pyramidal neurons have been classified into different subclasses based upon their spiking patterns (Franceschetti et al., 1998; Griego et al., 2022). We identified regularly spiking (RS) and intrinsic bursting (IB) pyramidal neurons in male and female BDNF^+/+^ and BDNF ^+/Met^ mice, which is consistent with others’ studies (Franceschetti et al., 1998; Griego et al., 2022; Hedrick and Waters, 2011). In male BDNF^+/+^ mice, 50% (8/16 neurons) of the recorded pyramidal neurons exhibited a regular spiking (RS) pattern in response to a series of depolarizing current steps and 50% (8/16 neurons) of them exhibited short bursts (two to five closely-spaced action potentials) (intrinsic bursting, IB) pattern. The proportion of these two types’ pyramidal neurons remained similar in female BDNF^+/+^ (RS, 56%, 9/16 neurons; IB, 44, 7/16 neurons), male BDNF ^+/Met^ (RS, 50%, 8/16 neurons; IB, 50%, 8/16 neurons) and female BDNF^+/Met^ mice (RS: 50%, 10/20 neurons; IB: 50%, 10/20 neurons). The input–output curve was conducted by gradually increasing the stimulus intensity of the depolarizing pulse (10 pA, 250 ms). The number of action potentials in regular spiking and intrinsic bursting pyramidal neurons was significantly increased with the incremented injected currents in male and female BDNF^+/+^ and BDNF^+/Met^ mice. The regular spiking and intrinsic bursting pyramidal neurons from male BDNF^+/Met^ mice displayed similar trends of higher number of evoked action potentials, but not significantly, compared to male and female BDNF^+/+^, and female BDNF^+/Met^ mice (Supplementary Figure S1). Then, we combined the regular spiking and intrinsic bursting pyramidal neurons together in each group. The number of evoked action potentials was significantly higher in the total pyramidal neurons of PFC from male BDNF^+/Met^ mice, but not female BDNF^+/Met^ mice, compared to male and female BDNF^+/+^ mice (*F*_Genotype (1, 64)_ = 3.7, *p* = 0.059, *F*_Sex (1, 64)_ = 2.8, *p* = 0.099, *F*_Genotype and Sex interaction (1, 64)_ = 5.4, *p* = 0.024, three-way ANOVA) (Figures 1B,C). Therefore, regular spiking and intrinsic bursting pyramidal neurons were pooled together in each group in this study. These results suggest that diminished activity-dependent BDNF signaling has a sexual dimorphic effect on the intrinsic excitability of pyramidal neurons in the PFC.

### 3.2 Diminished activity-dependent BDNF signaling significantly alters properties of action potential of pyramidal neurons in male mice, but not in female mice

**Figure 2:**
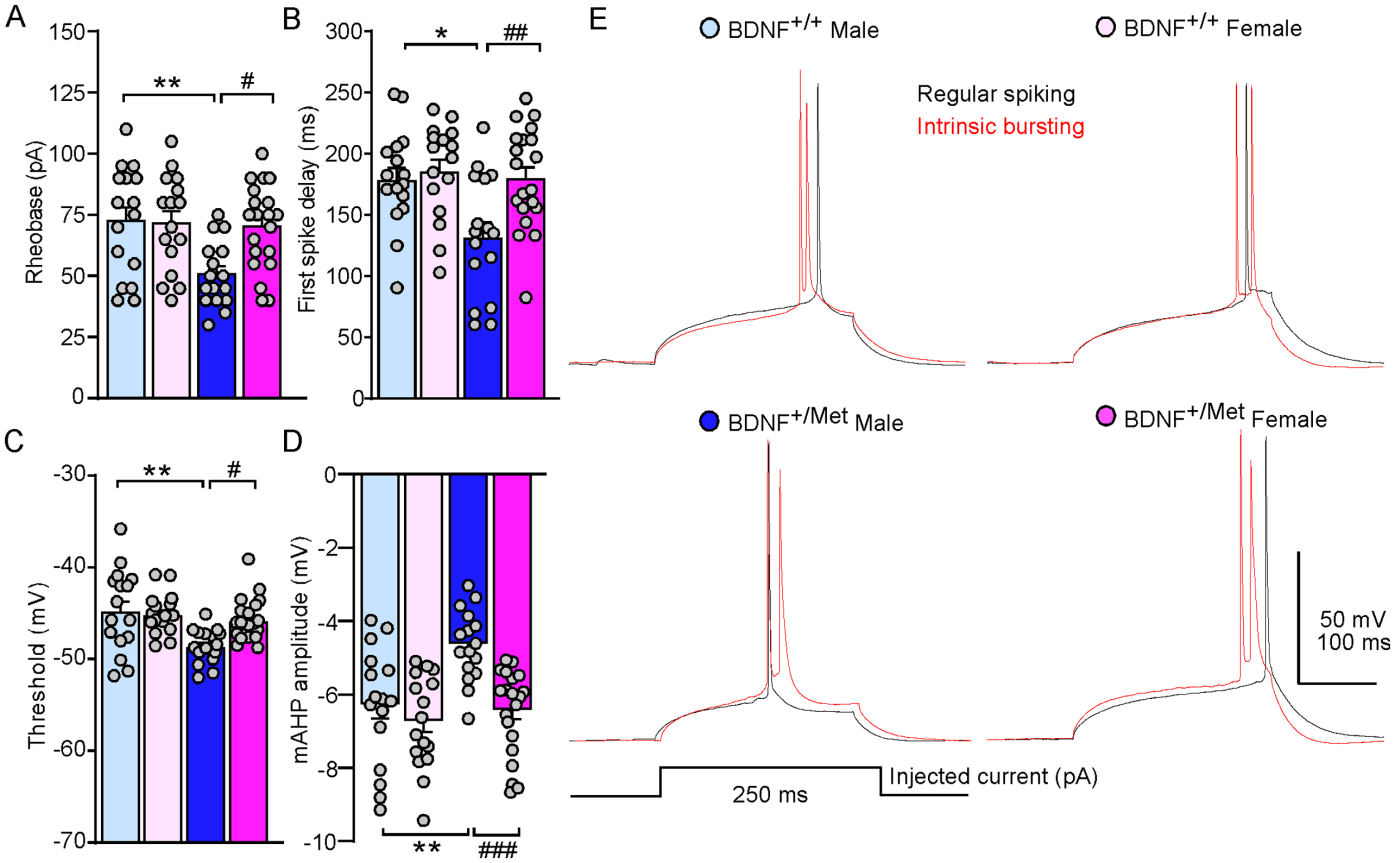
Diminished activity-dependent BDNF signaling differentially alters properties of action potential of pyramidal neurons in the PFC of males and females. Bar graphs showing rheobase **(A)**, first spike delay **(B)**, threshold **(C)**, and mAHP **(D)** in the pyramidal neurons of PFC from male and female BDNF^+/+^ and BDNF^+/Met^ mice. *p < 0.05, **p < 0.01, BDNF^+/Met^ versus BDNF^+/+^; #*p* < 0.05, ##*p* < 0.01, ###*p* < 0.001, male versus female, *n* = 16–20 neurons/4–5 mice/group, two-way ANOVA. **(E)** Representative traces of the first spike elicited with minimum electric current in regular spiking (black) and intrinsic bursting (red) pyramidal neurons.

To further determine the mechanisms of diminished activity-dependent BDNF signaling increased intrinsic excitability of pyramidal neurons in male mice, we examined the key parameters reflecting the action potential properties of pyramidal neurons in male and female BDNF^+/+^ and BDNF^+/Met^ mice. As shown in Figures 2A–E, diminished activity-dependent BDNF signaling significantly decreased rheobase in male, but not in female mice, the minimal electric current required to elicit an action potential when current was injected into a neuron (male BDNF^+/+^: 72.5 ± 5.6 pA; female BDNF^+/+^: 71.6 ± 4.9 pA; male BDNF^+/Met^: 50.6 ± 3.3; female BDNF^+/Met^: 70.3 ± 3.9 pA. n = 16–20 neurons/4–5 mice/group. *F*_Genotype (1, 64)_ = 6.7, *p =* 0.012; *F*_Sex (1, 64)_ = 4.3, *p* = 0.042; *F*_Genotype and Sex interaction (1, 64)_ = 5.2, *p* = 0.025, two-way ANOVA). Male BDNF^+/Met^ mice showed significantly shortened first spike latency, the period from the beginning of stimulus to the occurrence of first action potential (male BDNF^+/+^: 180.2 ± 10 ms; female BDNF^+/+^: 187.1 ± 9.8 ms; male BDNF^+/Met^: 133.2 ± 12.4 ms; female BDNF^+/Met^: 181.3 ± 9.2 ms. n = 16–20 neurons/4–5 mice/group. *F*_Genotype (1, 64)_ = 6.4, *p =* 0.01, *F*_Sex (1, 64)_ = 7.0, *p* = 0.01, *F*_Genotype and Sex interaction (1, 64)_ = 3.9, *p* = 0.05, two-way ANOVA). The threshold of action potential reflects how easily a neuron turns synaptic inputs into an action potential. The thresholds of action potentials were significantly lower in the pyramidal neurons from male BDNF^+/Met^ mice, but not from female mice, compared to male and female BDNF^+/+^ mice (male BDNF^+/+^: −44.6 ± 1.1 mV; female BDNF^+/+^: −45.0 ± 0.6 mV; male BDNF^+/Met^: −48.5 ± 0.5 mV; female BDNF^+/Met^: −45.7 ± 0.5 mV. n = 16–20 neurons/4–5 mice/group. *F*_Genotype (1, 64)_ = 10.4, *p* = 0.002, *F*_Sex (1, 64)_ = 2.9, *p* = 0.09, *F*_Genotype and Sex interaction (1, 64)_ = 5.2, *p* = 0.03, two-way ANOVA). Medium afterhyperpolarization (mAHP) affects the threshold of action potential and neuronal firing activity (Bean, 2007; Dwivedi and Bhalla, 2021). The amplitudes of mAHP were significantly smaller in the pyramidal neurons from male BDNF^+/Met^ mice, but not from female mice, compared to male and female BDNF^+/+^ mice (male BDNF^+/+^: −6.2 ± 0.4 mV; female BDNF^+/+^: −6.6 ± 0.3 mV; male BDNF^+/Met^: −4.5 ± 0.2 s; female BDNF^+/Met^: −6.3 ± 0.3 s. n = 16–20 neurons/4–5 mice/group. *F*_Genotype (1, 64)_ = 9.7, *p* = 0.003, *F*_Sex (1, 64)_ = 13.2, *p* = 0.0006, *F*_Genotype and Sex interaction (1, 64)_ = 4.7, *p* = 0.03, two-way ANOVA). These results demonstrate that diminished activity-dependent BDNF signaling differentially alters the properties of action potential in the pyramidal neurons from male and female mice, which mediates the sex effect of diminished activity-dependent BDNF signaling on the intrinsic excitability of pyramidal neurons.

### 3.3 Diminished activity-dependent BDNF signaling has no effects on passive membrane properties

The passive intrinsic properties of neurons are highly related to the ability of neurons to generate action potentials (Chen et al., 2020). Next, we examined the impact of diminished activity-dependent BDNF signaling on the passive membrane properties of pyramidal neurons in male and female mice. The passive membrane properties were determined by injecting a small hyperpolarizing current into the soma of neurons (100 pA). As shown in Supplementary Figure S2, there were no differences of resting membrane potentials, cell capacitance, input resistance, and Tau between male and female BDNF^+/+^ mice and BDNF^+/Met^ mice. These results demonstrated that diminished activity-dependent BDNF signaling has no effects on the passive membrane properties of pyramidal neurons in male and female mice, which indicates there are no significant differences in morphology of layer V pyramidal neurons in the PFC of male and female BDNF^+/+^ and BDNF^+/Met^ mice (Isokawa, 1997).

### 3.4 Diminished activity-dependent BDNF signaling increases the expression of *Scn2a* and voltage-gated sodium currents in male, but not in female mice

**Figure 3:**
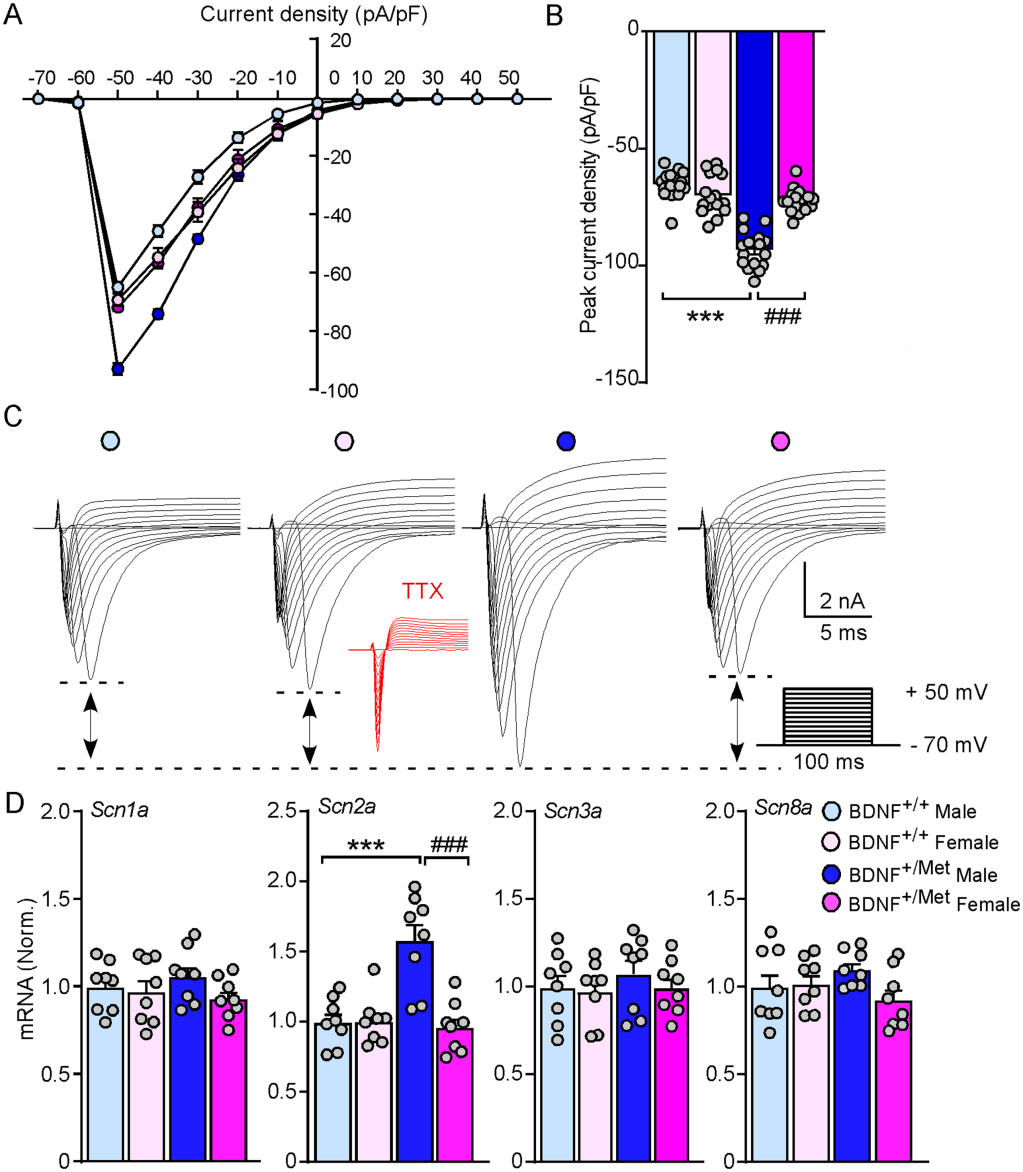
Diminished activity-dependent BDNF signaling significantly increases voltage-gated sodium currents and the expression of *Scn2a* in male mice, but not in female mice. **(A)** Current density-voltage relationship showing sodium current density in the pyramidal neurons from PFC of BDNF^+/+^ and BDNF^+/Met^ mice. *n* = 16 neurons/4 mice/group. **(B)** Bar graph showing the peak current density in the pyramidal neurons from PFC of BDNF^+/+^ and BDNF^+/Met^ mice. ***p < 0.001, BDNF^+/Met^ versus BDNF^+/+^; ###*p* < 0.001, male versus female, *n* = 16 neurons/4 mice/group, two-way ANOVA. **(C)** Representative traces of voltage-gated sodium currents recorded in response to voltage steps from −70 to +50 mV in the pyramidal neurons from PFC of BDNF^+/+^ and BDNF^+/Met^ mice. Inset: TTX (0.5 µM) abolished the inward currents. **(D)** Quantitative real-time PCR showing the mRNA levels of *Scn1a*, *Scn2a*, *Scn3a,* and *Scn8a* in the PFC of BDNF^+/+^ and BDNF^+/Met^ mice. ***p < 0.001, BDNF^+/Met^ versus BDNF^+/+^; ###*p* < 0.001, male versus female, *n* = 8 mice/group, two-way ANOVA.

Voltage-gated sodium channels play an essential role in generation and propagation of action potentials (Goldin et al., 2000). To determine whether diminished activity-dependent BDNF signaling increases the sodium currents in male mice, we evaluated the amplitudes of sodium currents in the pyramidal neurons of male and female BDNF^+/+^ and BDNF^+/Met^ mice in a voltage-clamp mode. The membrane potential was held at −70 mV. The total inward currents were recorded in response to voltage steps from −70 to +50 mV (10 mV step increase, 100 ms). As shown in current density curves and representative traces (Figures 3A–C), the peak inward currents were triggered at −50 mV. The inward currents were abolished in the presence of tetrodotoxin (TTX, 0.5 μM). The peak current densities were significantly increased in the pyramidal neurons from male BDNF^+/Met^ mice, compared to female BDNF^+/Met^ and BDNF^+/+^ mice (male BDNF^+/+^: −64.5 ± 1.5 pA/pF; female BDNF^+/+^: −68.9 ± 2.1 pA/pF; male BDNF^+/Met^: −92.5 ± 1.9 pA/pF; female BDNF^+/Met^: −71.4 ± 1.3 pA/pF, n = 16 neurons/4 mice/group, *F*_Genotype (1, 60)_ = 76.0, *p* < 0.0001; *F*_Sex (1, 60)_ = 22.9, *p* < 0.0001, *F*_Genotype and Sex interaction (1, 60)_ = 52.9, *p* < 0.0001). Among the 9 known members, Nav1.1, Nav1.2, Nav 1.3, and Nav1.6 are highly expressed in the central nervous system (Lai and Jan, 2006), which are composed by α and *β* subunits. To determine which sodium channel contributes to the decreased thresholds of action potentials in the pyramidal neurons from male BDNF^+/Met^ mice, we examined the transcriptional level of α subunits of these four sodium channels, given that the a subunit forms a pore that conducts sodium. As shown in Figure 3D, the mRNA levels of *Scn2a* (encoding Nav 1.2) were significantly higher in PFC lysates from male BDNF^+/Met^ mice (*F*_Genotype (1, 64)_ = 11.6, *p* = 0.002, *F*_Sex (1, 64)_ = 14.6, *p* = 0.0007, *F*_Genotype and Sex interaction (1, 64)_ = 15.4, *p* = 0.005), while the mRNA levels of *Scn1a* (encoding Nav 1.1), *Scn3a* (encoding Nav 1.3), and *Scn8a* (encoding Nav 1.6) were largely unchanged. These results suggest the increased *Scn2a* contributes to the higher sodium currents and decreased thresholds of action potentials in male BDNF^+/Met^ mice.

### 3.5 Diminished activity-dependent BDNF signaling selectively decreases the transcriptional level of *Kcnn2* in male mice, but not in female mice

**Figure 4:**
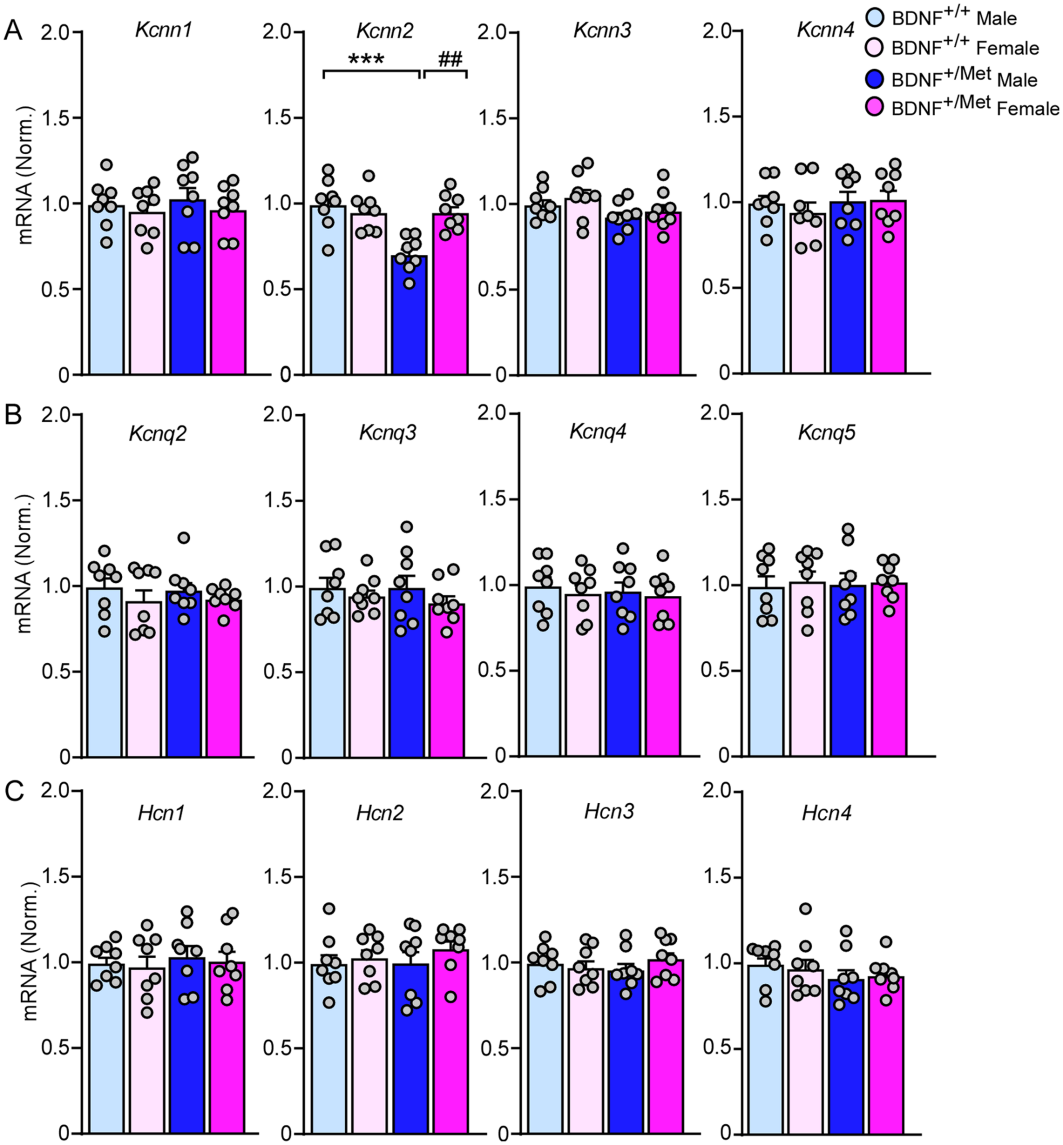
The impact of diminished activity-dependent BDNF signaling on the expression of SK channels, KCNQ channels, and HCN channels in the PFC. Quantitative real-time PCR showing the mRNA levels of SK channels **(A)**, KCNQ channels **(B)**, and HCN channels **(C)** in the PFC of BDNF^+/+^ and BDNF^+/Met^ mice. ***p < 0.001, BDNF^+/Met^ versus BDNF^+/+^; ##*p* < 0.01, male versus female, *n* = 8 mice/group, two-way ANOVA.

Medium afterhyperpolarization (mAHP) is mainly mediated by small conductance calcium activated potassium channels (SK), voltage-gated potassium channels 7 (KCNQ), and hyperpolarization activated cyclic nucleotide (HCN) channels (Dwivedi and Bhalla, 2021; Mateos-Aparicio et al., 2014; Gu et al., 2005). To find out which ion channels mediate the smaller mAHP in pyramidal neurons from male BDNF^+/Met^ mice (Figure 2E), we examined the transcriptional levels of *Kcnn1-4* (encoding SK1-4 channels), *Kcnq2-5* (encoding KCNQ channels), and *Hcn1-4* (encoding HCN1-4 channels) in the PFC. As shown in Figure 4, the mRNA levels of *kcnn2* (encoding SK2) were significantly decreased in PFC lysates from male BDNF^+/Met^ mice (*F*_Genotype (1, 28)_ = 12.5, *p* = 0.0015; *F*_Sex (1, 28)_ = 5.6, *p* = 0.025), while the mRNA levels of *Kcnn1*, *Kcnn3*, *Kcnn4, Kcnq2-5* and *Hcn1-4* were largely unchanged. These results suggest that the decreased *Kcnn2* is responsible for the smaller mAHP in male BDNF^+/Met^ mice.

## 4 Discussion

In this study, we characterized the impact of diminished activity-dependent BDNF signaling on the intrinsic excitability of pyramidal neurons in the PFC by using mice with knock-in of a human BDNF Met allele. Our results showed that diminished activity-dependent BDNF signaling increased intrinsic excitability of pyramidal neurons in male mice, but not in female mice. Furthermore, diminished activity-dependent BDNF signaling significantly increased the expression of *Scn2a* and decreased the expression of *Kcnn2* in male mice only, which decreased the thresholds of action potentials and facilitated the propagation of firing. We provide the first preclinical evidence that diminished activity-dependent BDNF signaling has a sexually dimorphic effect on the intrinsic excitability of pyramidal neurons in the PFC.

The high incidence of epilepsy in ASD patients indicates that the elevated excitability of pyramidal neurons is a major pathogenic factor in ASD (Blackmon et al., 2016; Sansa et al., 2011; Frye et al., 2013; Viscidi et al., 2013; Jeste and Tuchman, 2015). Increased intrinsic excitability of pyramidal neurons was found in the cortical neuronal culture by blocking neuronal activity by adding TTX, which was prevented by co-application of BDNF (Desai et al., 1999a; Desai et al., 1999b). In the present study, we found the evoked action potentials were significantly increased in the pyramidal neurons of PFC from male, but not female BDNF^+/Met^ mice, indicating the critical role of activity-dependent BDNF in homeostatic regulation of intrinsic excitability of pyramidal neurons. Additionally, the thresholds and mAHP of action potentials were significantly lower in male BDNF^+/Met^ mice. The higher AP frequencies, lower thresholds, and smaller mAHP of action potentials in male BDNF^+/Met^ mice indicate that activity-dependent BDNF differentially regulates intrinsic neuronal excitability in males and females.

One critical question is the underlying molecular mechanisms. The increased intrinsic excitability of pyramidal neurons in male BDNF^+/Met^ mice may occur through different ionic mechanisms. Voltage-gated sodium channels are one of the most important ion channels, which are crucial for the thresholds of action potentials. We found an upregulation of the sodium currents in pyramidal neurons of male BDNF^+/Met^ mice. Consistent with others’ report (Sontheimer and Ransom, 2002), the sodium currents showed abruptly activated large inward currents with voltage steps to −50 mV, which indicated intrinsic signs of poor voltage control in brain slices recordings. Activity deprivation increased the sodium currents without affecting activation nor inactivation characteristics of sodium channels (Desai et al., 1999b), which indicates diminished activity-dependent BDNF signaling will not alter the kinetic characteristics of sodium channels in male mice, though a voltage-independent change on open-channel probability cannot be ruled out (Li et al., 1992). Further studies are needed via minimizing the uncontrolled space-clamp axonal currents by applying a depolarizing pre-pulse before each voltage step (Milescu et al., 2010; Griego et al., 2022) or dissociated pyramidal neurons from PFC, given that somatic sodium currents are under good voltage control. Nav1.2 (encoded by *Scn2a*) is one of several sodium channels involved in initiation and propagation of action potentials in neurons (Sanders et al., 2018) and expressed predominantly in pyramidal cells (Spratt et al., 2019). The transcription of *Scn2a* is selectively upregulated in the PFC of male BDNF^+/Met^ mice. Nav1.2 is expressed in other cell types, such as interneurons (Yamagata et al., 2017), and glia cells (Pappalardo et al., 2016) in the PFC. The combined transcriptional and electrophysiological evidence strongly suggests the upregulated Nav1.2 mediates the increased sodium currents in the pyramidal neurons of male BDNF^+/Met^ mice.

mAHP is another parameter used to determine the intrinsic neuronal excitability, which is a hyperpolarized phase after a single or a train of action potentials and lasts 50 ~ 300 ms (Chen et al., 2020). mAHP is predominantly mediated by small conductance calcium-activated potassium (SK) channels, although voltage-gated potassium channels 7 (Kv7) and hyperpolarization-activated cyclic nucleotide-gated (HCN) channels have also been shown to contribute to the mAHP (Dwivedi and Bhalla, 2021; Gu et al., 2005).

SK channels are small-conductance calcium-activated potassium channels that are widely expressed in neurons and influence neuronal firing frequency (Faber and Sah, 2007; Sun et al., 2020). There are four family members in the SK channels, which are SK1, SK2, SK3, and SK4, encoded by *Kcnn1*, *Kcnn2*, *Kcnn3*, and *Kcnn4*, respectively. Activation of SK channels by calcium influx can modulate the frequency of action potential through rapid potassium efflux, which leads membrane repolarization/hyperpolarization. Alterations in SK channels have also been reported in various brain diseases. Humans carrying loss-of-function *KCNN2* mutations have been linked with ASD (Nam et al., 2023; Alonso-Gonzalez et al., 2019; Grove et al., 2019). Downregulation of SK2 was associated with augmented reticular thalamic bursting and seizures in *Scn1a*-Dravet syndrome (Ritter-Makinson et al., 2019). However, increased *Kcnn2* decreased intrinsic excitability of cortical pyramidal neurons in a PTEN-associated autism mouse model (Garcia-Junco-Clemente et al., 2013). Intellectual disability and developmental delay symptoms have been reported in patients carrying *KCNN3* variants (Bauer et al., 2019; Gripp et al., 2021; Schwarz et al., 2022). We found that diminished activity-dependent BDNF signaling selectively decreases *Kcnn2*, but *not Kcnn1*, *Kcnn3,* and *Kcnn4*.

*Kcnq* genes encode five family members of the Kv7 channels (Kv7.1–Kv7.5), which are a group of low-threshold voltage-gated potassium channels known as “M-channel” (Brown and Passmore, 2009). Four of them (*Kcnq2-5*) are expressed in the nervous system. Kv7.2 and Kv7.3 (encoded by *Kcnq2/3*) are the principal molecular components of M-channels, which are expressed on both excitatory and inhibitory neurons and constrain repetitive neuronal firing (Gu et al., 2005; Brown and Passmore, 2009; Delmas and Brown, 2005). Mutations of *KCNQ2/3* cause ASD and benign familial neonatal epilepsy (Satterstrom et al., 2020; Homozygosity Mapping Collaborative for Autism et al., 2014; Piro et al., 2019; Soldovieri et al., 2014; Maljevic and Lerche, 2014; Wang et al., 2020). *Kcnq2* deficiency in pyramidal neurons increased neuronal excitability, resulting in epilepsy (Peters et al., 2005; Soh et al., 2014). However, diminished activity-dependent BDNF signaling has no effect on the expressions of *Kcnq2-5*.

HCN channels are encoded by four genes (*Hcn1–4*), which can be activated typically at potentials below −50 mV and open at the resting membrane potential, conducting an inward cation current (*I*_h_) (Biel et al., 2009; Mishra and Narayanan, 2023; Santoro and Shah, 2020). In the mouse brain, HCN1 and HCN2 are predominantly expressed in the presynaptic synapse, soma, dendrites, and axon initial segments of layer V pyramidal neurons in the frontal cortex (Santoro and Shah, 2020), where they regulate glutamate release (Huang and Trussell, 2014) and generation of action potentials (Ko et al., 2016). The hyperexcitability of layer V pyramidal neurons in the anterior cingulate cortex was associated with a decrease in *I*_h_ (Santello and Nevian, 2015). *HCN1/2* are implicated in early infantile epileptic encephalopathy and absence seizures (Crunelli et al., 2023; Ludwig et al., 2003; Nava et al., 2014). In this study, there were no differences in the expressions of *Hcn1-4* between BDNF^+/+^ and BDNF^+/Met^ mice.

Therefore, diminished activity-dependent BDNF signaling selectively decreased the expression of *Kcnn2*, but *not Kcnn1*, *Kcnn3*, *Kcnn4*, Kv7 channels, and HCN channels, which indicates SK2 mediates the decreased mAHP in male BDNF^+/Met^ mice.

There are several limitations in this study. First, the causal link between upregulated *Scn2a,* decreased *Kccn2* and increased intrinsic excitatory in pyramidal neurons of male BDNF^+/Met^ mice is unknown. Selectively knockdown *Scn2a* and overexpression of *Kcnn2* in the pyramidal neurons of male BDNF^+/Met^ mice will confirm this causal link in our future studies. Female C57Bl/6 J mice exhibited higher glutamatergic transmission in the PFC compared to males (Knouse et al., 2022). Second, the contribution of synaptic inputs on the neuronal excitability of pyramidal neurons in the PFC, and the impact of diminished activity-dependent BDNF signaling on the principle neurons in the other brain regions, need to be further investigated, which may mediate the anxiety, autism-like social deficits, and sex-specific effect of diminished activity-dependent BDNF signaling on spatial memory (Ma et al., 2023). Third, the molecular mechanisms underlying the increased *Scn2a* and decreased *Kcnn2* in the PFC of male BDNF^+/Met^ mice are difficult to decipher at this moment, which will be investigated in future studies.

In summary, using a knock-in mouse model of the human BDNF Val66 Met SNP, the present study demonstrates that the increased *Scn2a* and decreased *Kcnn2* account for the sex effect of diminished activity-dependent BDNF signaling on the intrinsic excitability of pyramidal neurons in the PFC. The sex differences of intrinsic excitability of pyramidal neurons in the PFC of male and female BDNF^+/Met^ mice may partially contribute to the severe autism-like behavioral deficits in male BDNF^+/Met^ mice (Ma et al., 2023) and support that males are more vulnerable to having ASD (Giarelli et al., 2010; Werling and Geschwind, 2013). Targeting *Scn2a* and *Kcnn2* is a potential therapy for male ASD patients with or without BDNF Val66Met SNP.

## Data Availability

The raw data supporting the conclusions of this article will be made available by the authors, without undue reservation.

## Supplementary material

SUPPLEMENTARY FIGURE S1 Quantification of the number of evoked action potentials in response to injected currents in the regular spiking pyramidal neurons **(A)** and intrinsic bursting pyramidal neurons **(B)** of PFC from BDNF^+/+^ and BDNF^+/Met^ mice. A: *n* = 8-10 neurons/4-5 mice/group; B: n *=* 7-10 neurons/4-5 mice/group.

SUPPLEMENTARY FIGURE S2 Diminished activity-dependent BDNF signaling has no effect on the passive membrane properties of pyramidal neurons in male and female mice. *n* = 16-20 neurons/4-5 mice per group. **(A)** Resting potential, **(B)** Capacitance, **(C)** Input resistance, **(D)** Membrane time constant (τ), and **(E)** Representative traces in response to -100 pA hyperpolarizing current injection with 250 ms duration in the pyramidal neurons from PFC of BDNF^+/+^ and BDNF^+/Met^ mice.
