# Supplementary figures and images for: A sexually dimorphic signature of activity-dependent BDNF signaling on the intrinsic excitability of pyramidal neurons in the prefrontal cortex
Source: Front Cell Neurosci. 2024 Nov 6;18:1496930. doi: 10.3389/fncel.2024.1496930 (PMC11576208; doi:10.3389/fncel.2024.1496930)

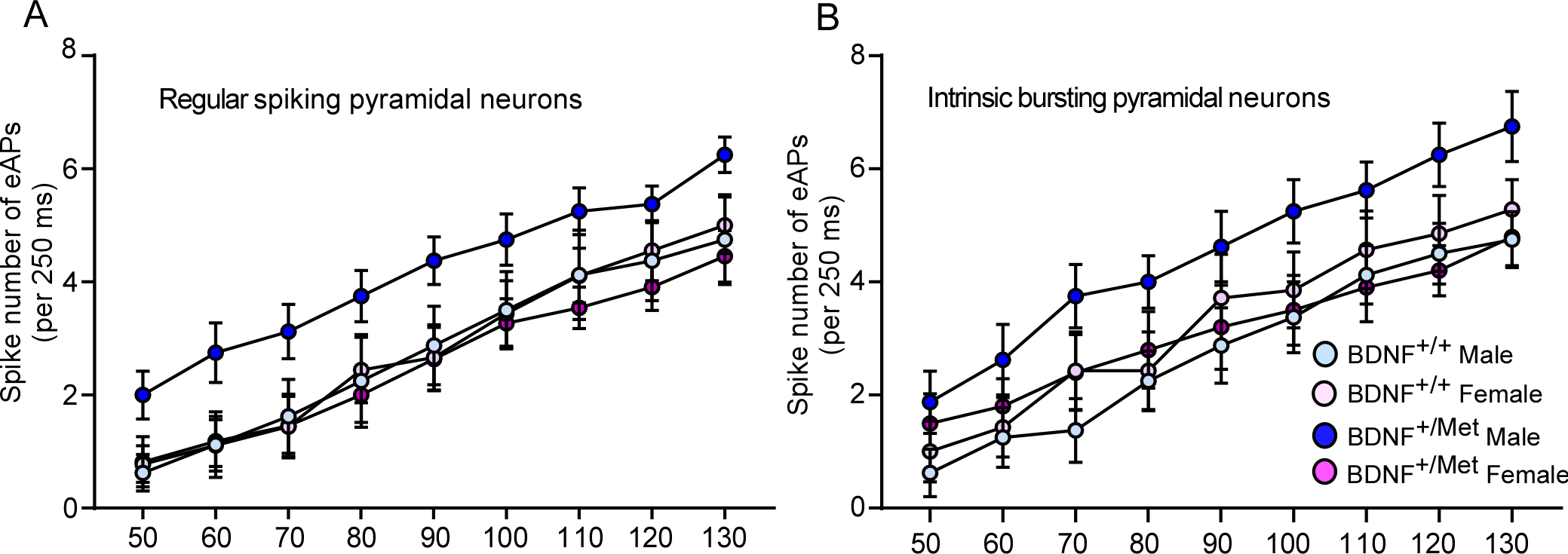

Supplement: SUPPLEMENTARY FIGURE S1 — Quantification of the number of evoked action potentials in response to injected currents in the regular spiking pyramidal neurons (A) and intrinsic bursting pyramidal neurons (B) of PFC from BDNF+/+ and BDNF+/Met mice. A: n = 8-10 neurons/4-5 mice/group; B: n = 7-10 neurons/4-5 mice/group. [file Image_1.TIF]

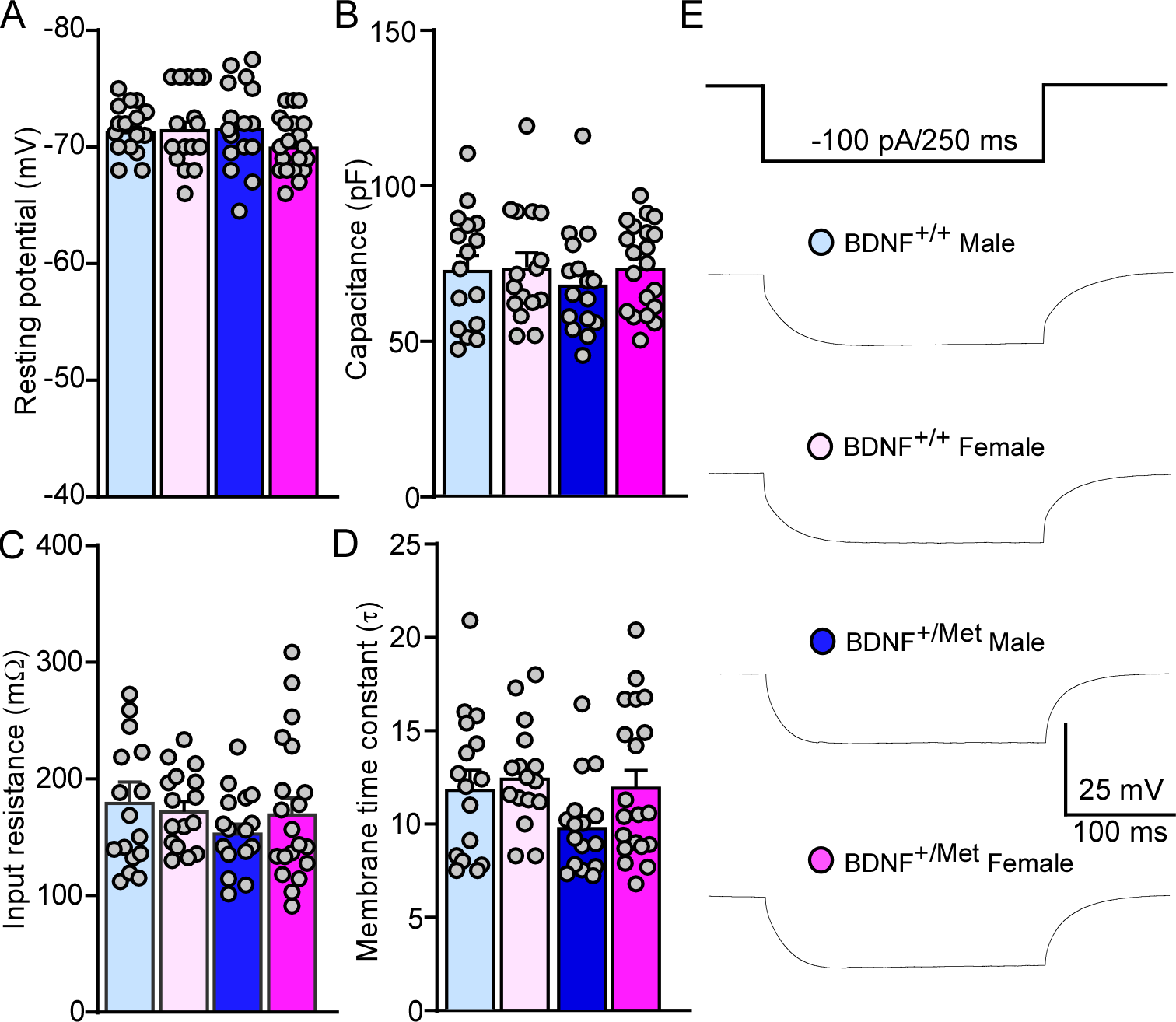

Supplement: SUPPLEMENTARY FIGURE S2 — Diminished activity-dependent BDNF signaling has no effect on the passive membrane properties of pyramidal neurons in male and female mice. n = 16-20 neurons/4-5 mice per group. (A) Resting potential, (B) Capacitance, (C) Input resistance, (D) Membrane time constant (τ), and (E) Representative traces in response to -100 pA hyperpolarizing current injection with 250 ms duration in the pyramidal neurons from PFC of BDNF+/+ and BDNF+/Met mice. [file Image_2.TIF]
